# Knowledge of and attitudes towards medical research ethics among first year doctoral students in Slovenia at the Faculty of Medicine, University of Ljubljana

**DOI:** 10.1186/s12909-023-04809-w

**Published:** 2023-11-03

**Authors:** S Grosek, D Pleterski Rigler, M Podbregar, V Erčulj

**Affiliations:** 1 Neonatology Section, Department of Perinatology, Division of Gynaecology and Obstetrics, University Medical Centre Ljubljana, Ljubljana, Slovenia; 2 Department of Medical Ethics, Faculty of Medicine, University of Ljubljana, Ljubljana, Slovenia; 3 National Committee of Medical Ethics of Republic Slovenia, Ljubljana, Slovenia; 4 Department of Internal Intensive Medicine, General Hospital Celje, Celje, Slovenia; 5 Department of Internal Medicine, Faculty of Medicine, University of Ljubljana, Ljubljana, Slovenia; 6 Rho Sigma Research & Statistics, Ljubljana, Slovenia; 7 Faculty of Criminal Justice and Security, University of Maribor, Ljubljana, Slovenia

**Keywords:** Research ethics, Integrity, Doctoral students, Biomedicine

## Abstract

**Background:**

Research ethics and attitudes should be the main concern of those who are conducting and publishing research in medicine.

**Methods:**

A cross-sectional study was conducted using a questionnaire among first year postgraduate doctoral students in Biomedicine at the Faculty of Medicine, University of Ljubljana during the academic year 2022/2023.

**Results:**

There were 54 out of 57 doctoral students included in the study, with a mean age (SD) of 29.7 (4.7) years, with predominantly female doctoral students, 66.7%. The number of correct answers out of 39 considered to illustrate students’ knowledge of medical research ethics was 31, meaning that they gave correct answers to 80% of all the questions. The mean number (SD) of correct answers was 18.9 (5.8), which significantly differed from 31 (*p* < 0.001). The previous experience of the doctoral students in research was significantly correlated with their knowledge of medical research ethics, even when controlling for the age, gender and workplace of respondents.

**Conclusion:**

This study clearly showed that insufficient knowledge and a poor level of attitudes exist about the main questions pertaining to medical research ethics. Overall knowledge is well below the expected positive answers. Further studies are needed to compare the knowledge of doctoral students with that of their tutors and what implications this might have for further teaching of research ethics.

**Supplementary Information:**

The online version contains supplementary material available at 10.1186/s12909-023-04809-w.

## Introduction

Planning and conducting research in medicine is inevitable in modern oriented *evidence-based medicine* if an improvement in treatment possibilities of more and more complex diseases is to be achieved [1–3]. However, research should be conducted by experienced as well as young researchers, who are or should be familiar with internationally agreed ethical principles and codes of integrity in research.

A long list of unethical human clinical trials in humans can be still extracted from the literature and media since research studies have begun to flourish, despite the sad history of unethical experimentation in the past. Despite many conscientious physicians and researchers alerted in the past about the occurrence of unethical experimentation in human subjects, it was not widely recognised until the Nuremberg trial, which showed the atrocities of German physicians in Nazi camps during the Second World War. The Nuremberg Code’s 10 points was part of the section of the judgement entitled “Permissible Medical Experiments”. It was only later that the Nuremberg Code became to some the most important document in the history of clinical research ethics [4]. Many of the critical points from the Nuremberg Code were expressed and cited in 1964 in the Declaration of Helsinki by the most prominent physicians in the World Medical Association. The Helsinki Declaration strongly emphasized the need for informed consent of participants in human research and ethical approval of such studies by research ethics committees, which should be established [5].

Despite international guidelines about research ethics, unethical behaviour in human research can be found daily in the media and scientific journals. To list only some of the most prominent: the Tuskegee Syphilis Study in the United States [6], Chinese researcher He Jiankui creating a gene-edited baby [7], Diederik Stapel, a Dutch social psychologist [8] deliberately misconducted studies over several years There are many others. In a study by Martinson et al. among American researchers in mid- and early-career, as many as 33% of researchers admitted to serious misconduct at least once during their scientific career [9].

A year before the Helsinki Declaration, in 1963 a Code of, now former, Yugoslav health professionals was adopted, in which it was mentioned that forced human experimentation is the worst violation of ethical principles, and human experimentation is only allowed if this medically and biologically justified and human subjects in experiments must be aware of the experiment and possible adverse effects and must voluntarily agree to participate [10].

In the Republic of Slovenia, ethics has been taught to undergraduate students at the Faculty of Medicine, University of Ljubljana since 1948 [11], and the first medical ethics committee was established in the second half of the1960s after the Declaration of Helsinki on human research came into effect [12]. This was among the first ethics committees in Europe, and its primary role was to evaluate the ethical adequacy of medical research projects by postgraduate doctoral students [11].

Teaching medical research ethics to undergraduate and postgraduate doctoral students at the Faculty of Medicine UL has a long tradition. In recent years, teaching for postgraduate students has even been strengthened to include 16 lectures covering all aspects of medical ethics and integrity in conducting human research. In 2022, we tried for the first time to evaluate knowledge of research ethics among Slovene doctoral students before they started lectures on research ethics and integrity. There is a scarcity of published research articles available at PubMed.gov under the key words: research ethics, postgraduate students, doctoral students [13, 14], which shows that knowledge and attitudes about research ethics is inadequate. In the first nationwide study on facing and solving ethical dilemmas among healthcare professionals in Slovenia in 2015 and in Croatia in 2016 when were studied the most frequent ethical dilemmas among health professionals, 5,3% and 11,0% participants answered that they face ethical dilemmas while conducting research at their university hospitals [15, 16].

We therefore hypothesized that knowledge among doctoral students *and* among those doctoral students who have had previous experience in conducting or participating in studies, with or without human subjects, is *not satisfactory.*

## Methods

### Study design

A cross-sectional study was performed during one afternoon, on Monday, November 14, 2022 at 16:00.

### Study participants

We recruited members of the first year postgraduate doctoral students (PhD students) in Biomedicine at the Faculty of Medicine, University of Ljubljana.

### Sampling method

The study aimed to include all students who were enrolled in the fall of 2022 in postgraduate doctoral study, i.e. 57 doctoral students. An online survey was conducted. The link to the questionnaire was sent by electronic mail to the students during the first hour of the course on medical ethics, which is part of the postgraduate course and consists of 16 lectures on different aspects of medical and research ethics and integrity. The PhD students were aware of this survey early, when they entered the postgraduate course. A questionnaire was sent to all students through their electronic mails, with access opened for the time of the study, i.e., 20 min, after which access was closed and blocked by November 14, 2022.

### Data collection tool

We developed a questionnaire based on our study objectives, that knowledge among doctoral students *and* also among those doctoral students who have had previous experience in conducting or participating in studies, with or without human subjects, is not satisfactory*.* All four authors prepared the questionnaire. The questionnaire, with 48 questions, consisted of 2 parts, with closed type questions (3 available answers; Yes; No; I don’t know). The first part related to general and demographic variables (age, gender, affiliation: surgical specialisations, internal specialisations, other medical specialisations (paediatrician, psychiatrist, neurologist and others) and other healthcare professions (biology, biochemistry, pharmacy, registered medical nurse or technician, veterinary medicine, psychology and so on), whether they had already been included in any research activities before or after graduation from their faculty and, if yes, what types of research activities. The last three questions of the survey were whether they had read the Tasks of the Medical Ethics Committee of Republic Slovenia (MEC RS), whether they know what informed consent is and whether they have ever submitted an application for ethics approval at MEC RS. The second part of the questionnaire (39 questions) explored the knowledge of doctoral students about research ethics (S1_File_Questionnaire).

The basis for preparing the questionnaire was an article written by Korošec and Trontelj (2003) on legislation related to research ethics in Slovenia, as a new country that was preparing to join the European Union [17]. This article is available online at the web site of MEC RS. They divided legislation issues into eight sections: 1. International Instruments in Slovene Legislation, 2. National Review, 3. Research on Humans, 4. Research on Biological Material of Human Origin (blood, organs, tissues, cells, DNA), 5. Research on Human Embryos and Embryonic Stem Cells, 6. Personal data, 7. Genetic Data, 8. Research on Animals. We prepared questions from each of these sections on the most relevant matters presented in each section related to research ethics, except for Sect. 7, for which questions were omitted, because we considered that this section is already partially covered in Sect. 4, and this section would need a special newly designed questionnaire due to evolving changes and constant new possibilities of processing genetic data which may generate possible misuses, especially regarding the protection of the unauthorized use of genetic material for new research without the new permission of the ethics.

### Statistics

Means and standard deviations were calculated for numerical, frequencies and percentages for categorical variables. The normality of the distribution of continuous variables was tested by the Shapiro–Wilk test. One sample t-test was used to test the difference of the mean number of correct answers from 31 (corresponding to 80% of correct answers). An independent sample t-test was used to test the difference in the mean number of correct answers between the group of students with previous experience with medical research and those without it. Univariate logistic regression or the likelihood ratio test were used to test the association between the correct reply to each question on medical research ethics and the study group. Multiple linear regression was used to test the relationship between the demographic variables, work-related variables and previous experience in research and the number of correct answers to questions concerning medical research ethics. *P* < 0.05 was considered statistically significant. SPSS version 28 was used for the statistical analysis.

## Results

There were 54 out of 57 doctoral students included in the study and their characteristics are summarized in Table 1. Three students were unavailable to complete the questionnaire at the time of survey; they were either on sick leave or unable to connect to website. The mean (SD) age of respondents was 29.7 (4.7) years. There were 18 (33.3%) men in the sample. The workplace of 20 (37%) students was internal medicine, 10 (18.5%) surgery, 22 (40.7%) another medical areas, such as paediatrics, dental medicine or oncology and 2 (3.7%) were studying veterinary medicine. Overall, 33 (61.1%) students had previous experience with medical research, mainly as part of a thesis at the 1^st^ Bologna level (64.5%).

**Table 1:** Sample description

	*n* = 54
Mean Age (SD)	29.7 (4.7)
Female gender; n (%)	46 (66.7)
Male gender; n (%)	18 (33.3)
Workplace	
Surgery	10 (18.5)
Internal medicine	20 (37)
Other medical specialisations	22 (40.7)
Other	2 (3.7)
Research project	33 (61.1)
1st Bologna level	
Student Prešeren thesis	20 (64.5)
Student research thesis	11 (35.5)
Diploma, master thesis	2 (6.5)
Other	5 (16.1)
2nd Bologna level	
Master before Bologna	1 (5.6)
Specialization thesis	3 (16.7)
ARRS project	6 (33.3)
Other	6 (33.3)
Previous ethical approval	26 (48.1)
Aware of informed consent	46 (85.2)
Read tasks of MEC	14 (25.9)

*Legend*: *MEC *Medical Ethics Committee, *ARRS *Slovenian Research Agency, *SD* Standard deviation

**Fig. 1:**
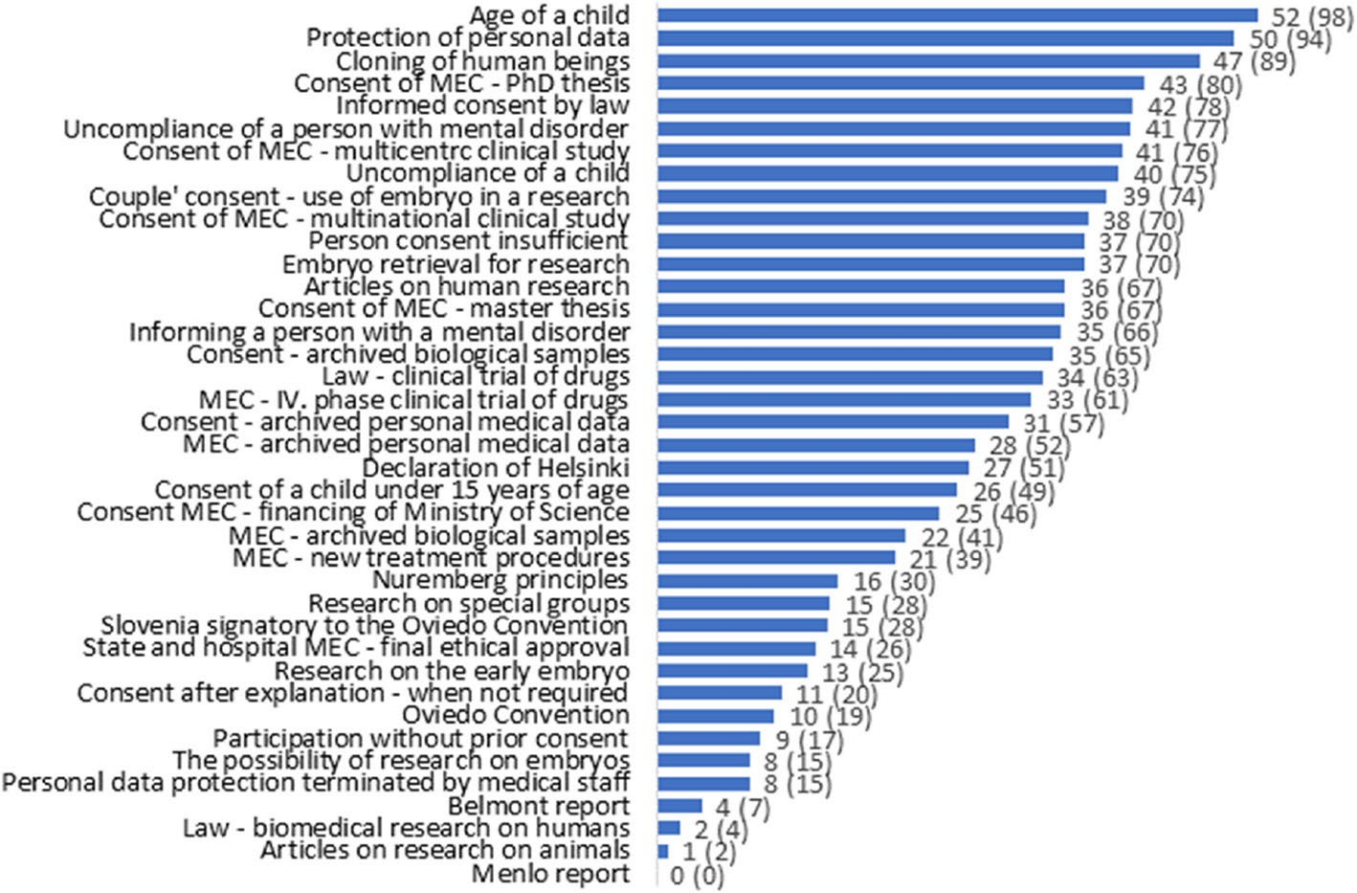
Frequency (%) of correct answers to questions relating to medical ethics (the columns represent percentages; MEC = Medical Ethics Committee)

Figure 1 illustrates the number and percentage of students giving the correct answer to each of the questions pertaining to medical ethics. Most students (52; 98%) knew that a child should be at least 15 years old to be able to give consent to participate in research and that retrieved personal data in medical research should be protected (50; 94%). Students also knew that cloning of human beings is not allowed in Slovenia (47; 89%) and that medical ethics committee approval is necessary even when doing research pertaining to a doctoral thesis (43; 80%). The least known topics on research ethics are the content of the Menlo report (none of the students answered correctly) or Belmont report (4 students answered correctly), whether the medical ethics code also includes articles on animals (only 1 student answered correctly) and whether biomedical research is regulated by law (2 students answered correctly).

**Fig. 2:**
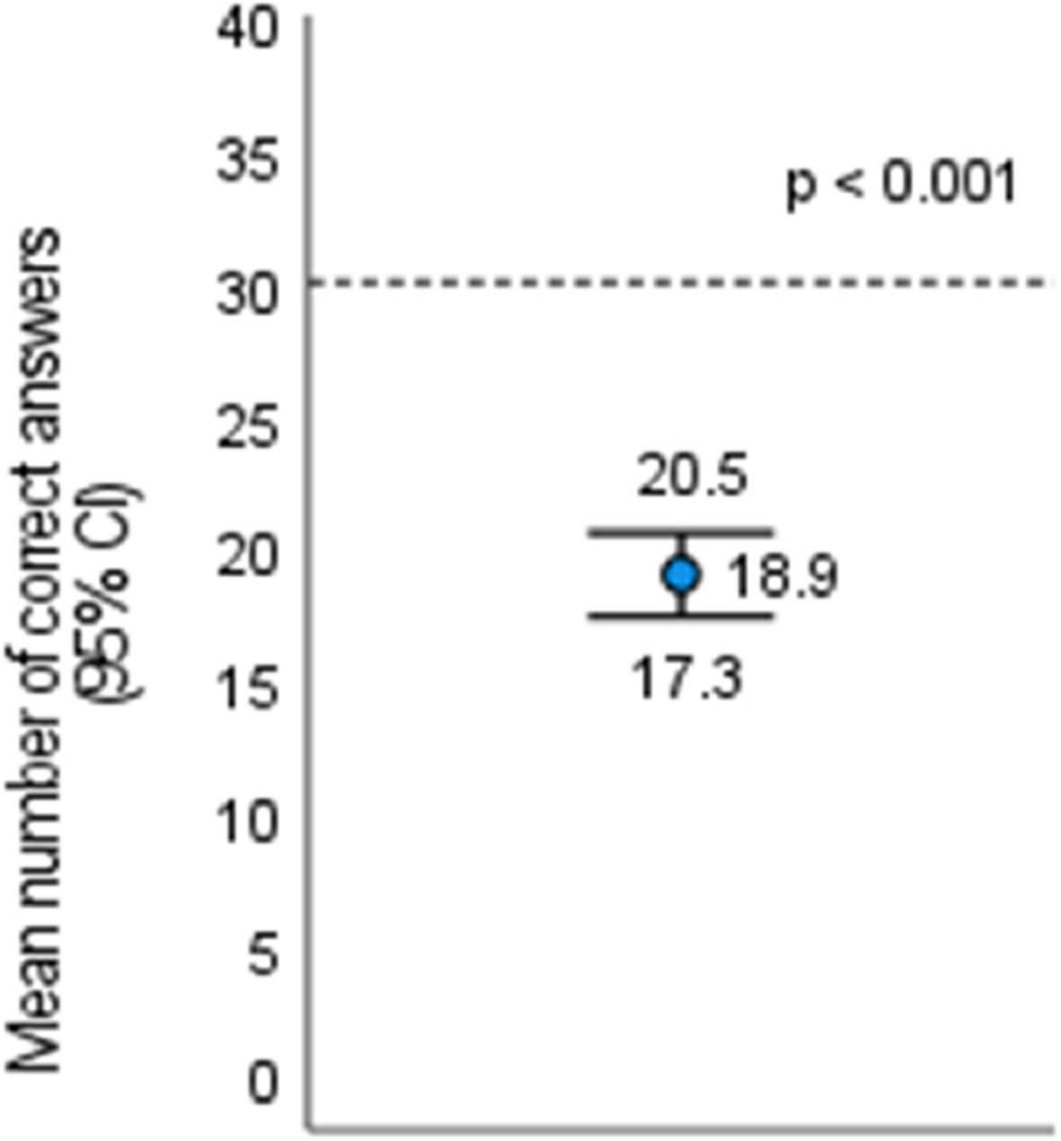
Mean and 95% CI of the number of correct answers

The mean number of correct answers with 95% CI is summarized in Fig. 2. The mean number (SD) of correct answers was 18.9 (5.8). The number of correct answers considered to illustrate students’ knowledge on medical ethics was 31; that means giving correct answers to 80% of all the questions. The obtained mean number of correct answers statistically significantly differed from 31 (*p* < 0.001).

Students with previous experience of medical research differed from those without experience in being knowledgeable about the content of the Helsinki Declaration (Table 2). They had ~ 5-times (95% CI: 1.4 – 15.8) higher odds of knowing the content of the Declaration than students without previous experience in research. They also had about 5-times (95% CI: 1.4 – 21.1) higher odds of answering correctly to the question about needing ethical approval when doing a multicentric clinical study. No other statistically significant associations between the number of correct answers and the group affiliation were found.

**Table 2:** Comparison of number (%) of correct answers between students conducting or helping in research with those without prior experience and results of logistic regression analysis

	No (*n* = 33)	Yes (*n* = 21)	OR (95% CI)	*P*
Nuremberg principles	4 (19)	12 (36.4)	2.43 (0.66—8.91)	0.181
Belmont report	2 (9.5)	2 (6.1)	0.61 (0.08—4.72)	0.638
Menlo report	0 (0)	0 (0)	1 (1; 1)	1
Declaration of Helsinki	6 (28.6)	21 (65.6)	4.77 (1.44—15.77)	0.01
Oviedo Convention	2 (9.5)	8 (25.8)	3.3 (0.63—17.45)	0.159
Slovenia signatory to the Oviedo Convention	3 (14.3)	12 (36.4)	3.43 (0.83—14.09)	0.087
Articles on human research	12 (57.1)	24 (72.7)	2 (0.63—6.35)	0.239
Articles on research on animals	0 (0)	1 (3)		0.318*
Law—biomedical research on humans	0 (0)	2 (6.1)		0.155*
Law—clinical trial of drugs	13 (61.9)	21 (63.6)	1.08 (0.35—3.34)	0.898
Consent of MEC—PhD thesis	15 (71.4)	28 (84.8)	2.24 (0.59—8.58)	0.239
Consent of MEC—Master thesis	13 (61.9)	23 (69.7)	1.42 (0.45—4.48)	0.554
Consent MEC—financing of Ministry of Science	8 (38.1)	17 (51.5)	1.73 (0.57—5.26)	0.337
Consent of MEC—multicentric clinical study	12 (57.1)	29 (87.9)	5.44 (1.4—21.11)	0.014
Consent of MEC—multinational clinical study	13 (61.9)	25 (75.8)	1.92 (0.59—6.3)	0.28
State and hospital MEC—final ethical approval	3 (14.3)	11 (33.3)	3 (0.72—12.42)	0.13
MEC—new treatment procedures	7 (33.3)	14 (42.4)	1.47 (0.47—4.61)	0.505
Informed consent by law	14 (66.7)	28 (84.8)	2.8 (0.75—10.43)	0.125
Consent—archived personal medical data	10 (47.6)	21 (63.6)	1.92 (0.63—5.85)	0.248
MEC—archived personal medical data	10 (47.6)	18 (54.5)	1.32 (0.44—3.95)	0.62
Consent—archived biological samples	14 (66.7)	21 (63.6)	0.88 (0.28—2.77)	0.82
MEC—archived biological samples	9 (42.9)	13 (39.4)	0.87 (0.29—2.63)	0.801
Consent after explanation—when not required	4 (19)	7 (21.2)	1.14 (0.29—4.51)	0.847
MEC—phase IV clinical trial of drugs	12 (57.1)	21 (63.6)	1.31 (0.43—4.01)	0.634
Person’s consent insufficient^a^	13 (61.9)	24 (75)	1.85 (0.56—6.07)	0.313
Participation without prior consent^a^	6 (28.6)	3 (9.4)	0.26 (0.06—1.18)	0.081
Age of a childa^a^	21 (100)	31 (96.9)		0.312*
Consent of a child under 15 years of age^a^	10 (47.6)	16 (50)	1.1 (0.37—3.31)	0.865
Non-compliance of a child^a^	13 (61.9)	27 (84.4)	3.32 (0.91—12.18)	0.07
Informing a person with a mental disorder^a^	13 (61.9)	22 (68.8)	1.35 (0.43—4.3)	0.607
Non-compliance of a person with a mental disorder^a^	14 (66.7)	27 (84.4)	2.7 (0.72—10.07)	0.139
Research on special groups^a^	3 (14.3)	12 (37.5)	3.6 (0.87—14.84)	0.076
Research on early embryo^a^	4 (19)	9 (28.1)	1.66 (0.44—6.31)	0.455
Possibility of research on embryos^a^	0 (0)	8 (25)		0.003*
Couple's consent—use of embryo in research^a^	15 (71.4)	24 (75)	1.2 (0.35—4.15)	0.773
Embryo retrieval for research^a^	12 (57.1)	25 (78.1)	2.68 (0.8—8.93)	0.109
Cloning of human beings^a^	17 (81)	30 (93.8)	3.53 (0.58—21.32)	0.169
Protection of personal data^a^	19 (90.5)	31 (96.9)	3.26 (0.28—38.48)	0.348
Personal data protection terminated by medical staff^a^	3 (14.3)	5 (15.6)	1.11 (0.24—5.24)	0.894

*Legend*: *Likelihood ratio test; *MEC *Medical Ethics Committee, PhD thesis-doctoral thesis; ^a^The sample size for the group of students with previous research reduced to *n* = 20

**Fig. 3:**
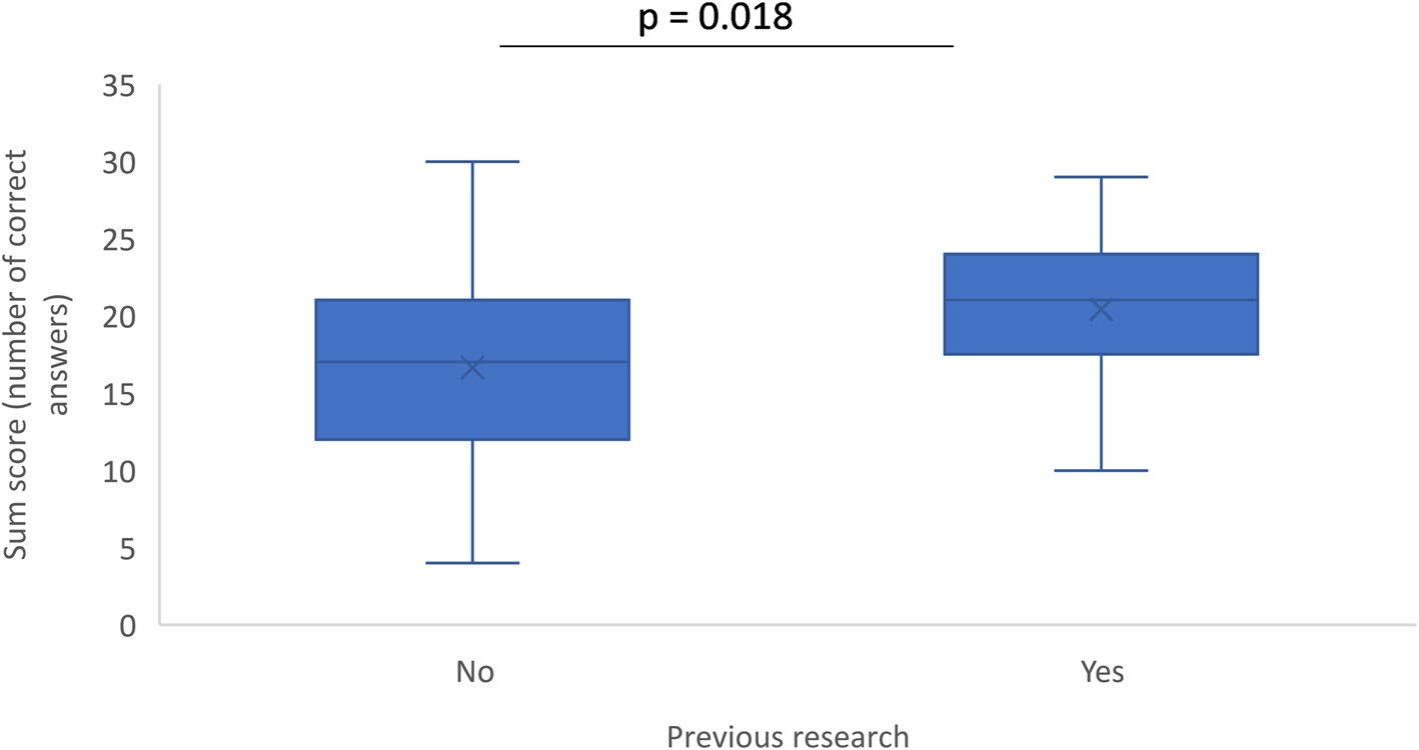
Box plots of the number of correct answers by study group

A comparison of the number of correct answers between the two study groups showed a statistically significant difference (*p* = 0.018; Fig. 3).

Previous experience in research was statistically significantly correlated with knowledge of medical research ethics, even when controlling for age, gender and workplace of respondents (Table 3).

**Table 3:** Relationship between demographic and work-related variables and the number of correct answers (results of multiple linear regression)

	Std. reg. Coeff	*P*
Age	0.020	0.869
Male	-0.110	0.394
Surgeon vs Other	0.220	0.135
Internal medicine vs. Other	0.120	0.411
Previous research experience	0.300	0.028

R^2^ = 0.16

## Discussion

This is the first Slovene study on ethical knowledge and attitudes of medical research ethics among postgraduate doctoral students of Biomedicine at the Faculty of Medicine, University of Ljubljana. This study clearly showed insufficient knowledge and unsatisfactory level of attitudes about the main questions pertaining to medical research ethics. Overall knowledge was well below the expected positive answers, which should have been 80% or above. Previous experience in research among the participants was statistically significantly related to knowledge of medical research ethics, even when controlling for age, gender and workplace of respondents.

As already said, the basis for preparing the questionnaire was an article written by Korošec and Trontelj on legislation related to research ethics in Slovenia, as a new country that was preparing to join the European Union, and which is available on the web site of MEC RS [17]. We were specifically interested in getting answers about seven topics; International Instruments in Slovenian Legislation, National Review, Research on Humans, Research on Human Embryos and Embryonic Stem Cells, Research on Biological Material of Human Origin, Research on Animals, and Personal Data.

Undergraduate medical studies in Slovenia have a strong emphasis on the deontological part of ethics and international instruments related to human research, such as Nuremberg, the Helsinki Declaration, the Oviedo report, and other codes, principles and declarations, but despite that previous teaching, positive answers to these questions were scaled the worst, on the lower half or bottom of the scaling (see Fig. 1).

One of the important issues in the ethics of medical research is for doctoral students to have enough knowledge before conducting their research projects. Young et al. showed in their study that knowledge of the principles of research ethics and that knowledge of basic research ethics concepts, including the Helsinki Declaration (87.5%) was good, but almost half lower than knowledge of the Nuremberg Code (52.4%) [18]. In present study knowledge of the Helsinki Declaration was positively answered by only 51% and of the Nuremberg principles by only 30%. Furthermore, when we tested these results between two groups of students, those with and those without previous experience of conducting or helping in research, those with prior experience had almost 5-times higher statistically significant odds of knowing the content of the Helsinki Declaration and 2.4-times higher odds for the Nuremberg principles, though this was not statistically significant. These results shows a great opportunity to improve knowledge of the both groups of doctoral students. Informed consent was extremely important for participants in the Young study, with 100% of them answering positively to that question, However, in practice, informed consent was obtained in only 52.4% of cases [18]. In our study, 78% of the doctoral students answered that informed consent is required by law. When we asked whether informed consent is required for specific situations, i.e., when informed consent is not sufficient (70%) or needed for archived biological samples (65%), archived personal medical data (57%), when consent is not required (20%) and when participation can proceed without prior consent (17%), the positive answers were much lower. Results on national review, research on humans, research on human embryos and embryonic stem cells showed a dichotomous distribution, i.e., the answers of participants to some questions from these three topics were very good, while for some worse. However, a high percentage of participants knew that cloning of human beings and embryo retrieval only for research is not allowed, although they did not know whether research on early embryos is allowed or not in Slovenia, and if yes, with what exemptions.

In Europe, a recent study by Abdi et al. among members of the League of European Research Universities (LERU) to map out the content, format, frequency, duration, timing and compulsory status of their training programmes and the characteristics of instructors of onsite courses, revealed substantial variation in educational materials among the studied institutions [19]. The European Code of Conduct for Research Integrity specifies that good research practices are based on basic principles of research integrity, which are intended to guide researchers in their work. The most important principles quoted are reliability, honesty in the development of all phases of research, respect for colleagues and responsibility for research from conception to publication (ALLEA 2018) [20]. Research institutions and organizations ensure that researchers receive thorough training in research design, methodology and analysis [21]. American philosophers, in the book Principles of Medical Ethics, wrote about moral virtues that should be inherent to medical professionals, and the same is also true for researchers. Among the five moral virtues for them, the most important is professional integrity, i.e., in research, research integrity [22]. Because moral virtues, e.g., integrity, can be taught, it is important to educate researchers about research integrity.

The current study has some strengths, being the first in Slovenia comprehensively to tackle the issue of knowledge and attitudes of doctoral students about research ethics. It clearly has the limitation of being done in only one centre and having a relatively small number of participants. A future study with next set of doctoral students is justified, which should include doctoral tutors before and after classes on research ethics in the postgraduate study of Biomedicine.

This means that before being involved in conducting studies, doctoral students should have passed a test of knowledge, attitudes and integrity. Lack of knowledge of research ethics may lead to misconduct of researchers and a lack of integrity. However, despite the results of the presented study, the national MEC receives ethically prepared doctoral theses, which is probably a reflection of the adequate work of the mentors of the doctoral students.

### Supplementary Information

**Additional file 1.** 

**Additional file 2.**

## Data Availability

The datasets used and/or analyzed during the current study are available from the corresponding author on reasonable request.
