# Supplementary material for: Knowledge of and attitudes towards medical research ethics among first year doctoral students in Slovenia at the Faculty of Medicine, University of Ljubljana
Source: BMC Med Educ. 2023 Nov 3;23:828. doi: 10.1186/s12909-023-04809-w (PMC10623751; doi:10.1186/s12909-023-04809-w)
Supplement: Supplementary file 1 — Additional file 1. [file 12909_2023_4809_MOESM1_ESM.docx]

**Supporting information**

**Title of the S1_File:**

**»Knowledge of and attitudes to some ethical issues in the research work of doctoral students of the Biomedicine Program at the Faculty of Medicine, University of Ljubljana”**

To whom it may concern!

Knowledge is essential in research work, not only of technical and professional requirements, but also of ethical issues that arise and how to respond to them, as well as the attitudes you have when ethical issues do arise in research work.

In order to assess your knowledge and attitudes towards basic ethical principles in research work, we are asking you to fill out the questionnaire attached below. The questionnaire is completely anonymized, and it will help us in preparing the contents of doctoral study in Biomedicine in the future, as well as in the undergraduate study of Medicine and Dental Medicine at the Medical Faculty of the University of Ljubljana.

We would also like to prepare an article from the survey results and we ask that by voluntarily participating in this research, you agree to the processing of anonymized data for the purpose of the article and its publication. Where the masculine form of a profession or person is written, it also applies to the feminine form (e.g., biologist, colleague etc...). Voluntary completion of the questionnaire means that you agree to be included in the research and the publication of the research results in the professional literature.

The authors of the questionnaire thank you very much for your cooperation.

Prof. Dr. Štefan Grosek, M.D.

Prim. Dušica Pleterski Rigler, M.D.

Prof. Dr. Matej Podbregar, M.D.

Doc. Dr. Vanja Erčulj, B.Sc. (Psychology).

**DEMOGRAPHIC DATA**

Age (enter age in years): ............years

Sex: M

W

Position and/or specialisation (circle the answer):

a. surgical profession (all surgical professions):

b. internal medicine profession (all internal medicine professions)

c. other medical professions:

d. other (biologist, biochemist, psychologist, pharmacist, registered nurse, registered medical doctor, other - enter occupation:...............)

I have already cooperated in a research project:

NO, this is the first research

If YES, enter whether undergraduate and/or postgraduate below (more than one answer is possible)

A. Undergraduate:

a. Prešeren student assignment

b. other (diploma thesis, master thesis - Bologna system)

c. student research paper

d. other

**B. Postgraduate:**

a. master thesis,

b. specialist task

c. associate of research program at ARRS

d. collaborator in international research program

e. other (enter where):..........................................

KNOWLEDGE AND ATTITUDES (circle the answer)

1. Before starting your doctoral research project, have you read the powers and tasks of the Medical Ethics Commission of the Republic of Slovenia (MEC RS)?
2. YES
3. NO
4. DON'T KNOW

2. Do you know what informed consent to research is?

1. YES
2. NO
3. DON'T KNOW
4. Have you ever lodged an application for an assessment of the ethicality of a research task at MEC RS?
5. YES
6. NO
7. Are you familiar with the Nuremburg principle in research?
8. YES
9. NO
10. DON'T KNOW
11. If YES, what is the most important theme dealt with?
    1. Involuntariness
    2. Compulsion
    3. Consent to research after instruction on the protection of human rights
12. Are you familiar with the Belmont Report?
    1. Yes
    2. NO
    3. DON'T KNOW
13. If YES, what are its three most important characteristics:
    1. respect for persons, charity, justice
    2. autonomy, harmlessness, injustice
    3. harmfulness, injustice, equality
14. Are you familiar with the Menlo report?
    1. YES
    2. NO
    3. DON'T KNOW
15. If YES, what is its fourth important characteristic in addition to the three respects: for personality, charity and justice?

a. harmlessness

b. respect for privacy

c. respect for law and the public interest

1. Are you familiar with the Helsinki Declaration?
   1. YES
   2. NO
   3. DON'T KNOW
2. If YES, what are its most important characteristics:

a. autonomy and safety of persons involved in research, informed consent to research, identification of vulnerable groups

b. harmlessness, unfairness, holisticness

c. harmlessness, injustice and charity

12. Are you familiar with the Oviedo Convention?

- 1. YES
  2. NO
  3. DON'T KNOW

1. If YES, is Slovenia a signatory?
   1. YES
   2. NO
   3. DON'T KNOW
2. If YES, do you know what are the main principles?

a. protection of persons in research, voluntariness and informed consent to the research, care for vulnerable groups

b. no detailed instruction is required prior to consent, since a lay person cannot understand technical terms

c. minors may not give consent

15. Does the Slovene Code of Medical Ethics also contain articles on human research?

- 1. YES
  2. NO
  3. DON'T KNOW

1. Does the Slovene Code of Medical Ethics also contain articles on animal research?
   1. YES
   2. NO
   3. DON'T KNOW
2. Do you know whether Slovenia has legislation regulating biomedical research on humans?
3. YES
4. NO
5. DON'T KNOW
6. Does Slovenia have legislation on clinical research of medicines?
7. YES
8. NO
9. DON'T KNOW
10. Must all research as part of a doctoral thesis at the MF obtain the consent of MEC RS?
    1. YES
    2. NO
    3. DON'T KNOW
11. Must all research as part of a master thesis at the MF obtain the consent of MEC RS?
12. YES
13. NO
14. DON'T KNOW
15. Must all research financed by the ministry responsible for science obtain the consent of MEC RS?
16. YES
17. NO
18. DON'T KNOW
19. Must all multicentric clinical research obtain the consent of MEC RS?
20. YES
21. NO
22. DON'T KNOW
23. Must all multinational clinical research obtain consent from MEC RS?
    1. YES
    2. NO
    3. DON'T KNOW
24. Do the ethics commission of the Institute of Oncology, the State Commission for Fertilization with Biomedical Assistance, hospital MECs’ make a final decision on the ethical suitability of research?
25. YES
26. NO
27. DON'T KNOW
28. Is MEC RS authorised to assess the ethical acceptability of new treatment procedures?
29. YES
30. NO
31. DON'T KNOW
32. Does Slovene legislation require informed consent to research?
33. YES
34. NO
35. DON'T KNOW
36. Is consent required for research use of archived personal medical data?
37. YES
38. NO
39. DON'T KNOW
40. Is consent required for research use of archived biological samples?
    1. YES
    2. NO
    3. DON'T KNOW
41. Can MEC RS release an applicant from the obligation to obtain consent to the research use of archived biological samples?
    1. YES
    2. NO
    3. DON'T KNOW
42. Can MEC RS release an applicant from the obligation to obtain consent to the research use of archived personal data?

a. YES

b. NO

c. DON'T KNOW

32. When is informed consent not required?

a. it is always required

b. it is not required if the data is anonymous

c. it is not required if there is no possibility of violation of human rights

33. Do phase IV clinical trials of drugs need an assessment by MEC RS?

1. YES
2. NO
3. DON'T KNOW
4. When is a person's consent to participate in research not sufficient?

a. if the person is not capable of making decisions about himself

b. if he is paid for his participation

c. if a child is under 15 years old

35. When can people participate in research without prior consent?

a. if the situation is urgent and the person cannot give informed consent

b. even if the situation is urgent, a person may not be included in research if, prior to this, they did not give informed consent

1. DON’T KNOW
2. At what age are children by law capable of giving consent for cooperation in research (circle the right answer)? At age:
3. 11 years
4. 13 years
5. 15 years
6. Can a child under 15 years of age give consent for inclusion in research?
7. YES
8. NO
9. DON'T KNOW
10. If a child opposes a proposed protocol of research?
11. the research may not be started or continued
12. the start may be aborted and a second attempt made
13. DON'T KNOW
14. If a person has a mental disorder, the research must be explained to the maximum extent permitted by their condition and consent to participate in the research must be obtained based on their will
15. YES
16. NO
17. DON'T KNOW
18. If a person with a mental disorder opposes participation in research?
    1. the consent of their guardian is sufficient for the inclusion of the person opposing it
    2. the person who opposes it may not be included in the research
    3. DON'T KNOW
19. When can research on special groups, e.g., pregnant women, be conducted? (multiple answers are possible)
    1. it can always be conducted
    2. if results cannot be obtained on animals or persons who do not belong to these sensitive groups
    3. if the risk is minimal in relation to possible real and direct benefits of the research
20. Is early embryo research possible in Slovenia?
21. YES
22. NO
23. DON'T KNOW
24. Research is permitted on embryos which (multiple answers are possible):
    1. are not suitable for insertion in a woman's body
    2. are not suitable for storage
    3. are at least 5 years old
    4. embryo research is not permitted
25. Is the written consent of a couple required for research on embryos with which fertilisation has been assisted by a biomedical procedure and some early embryos are left over or are in storage?
26. YES
27. NO
28. DON'T KNOW
29. Is it permitted in Slovenia to obtain embryos purely for the purpose of research?
30. YES
31. NO
32. DON'T KNOW
33. Is cloning human beings permitted in Slovenia?
34. YES
35. NO
36. DON'T KNOW
37. Is it necessary to protect personal data (identity data, health data) in research?
38. YES
39. NO
40. DON'T KNOW
41. In what cases can a doctor/medical worker/collaborator terminate the protection of personal data?
    1. for the protection of their own treatment procedures or research participation
    2. when disclosed or permitted to be disclosed by a patient or research subject
    3. DON'T KNOW

Thank you for your time and the effort that you have invested in answering the questionnaire.

Date and signature:

Prof. Dr. Štefan Grosek, M.D.
